# RBMS2 inhibits the proliferation by stabilizing P21 mRNA in breast cancer

**DOI:** 10.1186/s13046-018-0968-z

**Published:** 2018-12-04

**Authors:** Xi Sun, Yue Hu, Jing Wu, Liang Shi, Lei Zhu, Pei-Wen Xi, Ji-Fu Wei, Qiang Ding

**Affiliations:** 1 Jiangsu Breast Disease Center, the First Affiliated Hospital with Nanjing Medical University, 300 Guangzhou Road, Nanjing, 210029 China; 2 Research Division of Clinical Pharmacology, the First Affiliated Hospital with Nanjing Medical University, 300 Guangzhou Road, Nanjing, 210029 China

**Keywords:** Breast cancer, RBMS2, P21, Tumor suppressor

## Abstract

**Background:**

RNA binding proteins (RBPs) play an important role in regulating the metabolism of target RNAs. Aberrant expression of RBPs plays a vital role in the initiation and development of many cancers. The RBM family, which has the conserved RNA binding motif RNP1 and RNP2, shares the similar function in RNA processing and RBMS2 is a member of them. P21, also named CDKN1A, promotes cell cycle arrest and plays an important role in halting cell proliferation. In our study, we identified RBMS2 as a tumor suppressor in breast cancer. It inhibited the proliferation of breast cancer by positively regulating the stability of P21 mRNA in posttranscriptional way.

**Methods:**

TCGA was used to identify differentially expressed RBPs in breast cancer.

The effect of RBMS2 on breast cancer proliferation was evaluated in vitro using CCK-8 assays, colony formation assays and cell-cycle assays and the in vivo effect was investigated using a mouse tumorigenicity model.

The main pathway and genes regulated by RBMS2 was detected by RNA sequencing. The RNA immunoprecipitation combined with dual-luciferase reporter assay were conducted to testify the direct binding between RBMS2 and P21. Rescue assay was used to detect P21 as the main target of RBMS2.

**Results:**

The expression of RBMS2 was lower in breast cancer compared with normal tissues and was a favorable biomarker in breast cancer. RBMS2 inhibited the proliferation of breast cancer and P21 was the main target of RBMS2. RBMS2 stabilized the mRNA of P21 by directly binding to the AU-rich element of 3′-UTR region. Anti-proliferation activity induced by overexpression of RBMS2 was rescued by interfering with the expression of P21.

**Conclusion:**

In conclusion, RBMS2 acted as a tumor suppressor in breast cancer and positively regulated the expression of P21 by stabilizing its mRNA.

**Electronic supplementary material:**

The online version of this article (10.1186/s13046-018-0968-z) contains supplementary material, which is available to authorized users.

## Introduction

As the most common cancer among women, breast cancer is expected to account for 30% of all new cancer diagnoses in women. It has posed a great threat to world health as the second leading cause of cancer death among women [1]. The death rates of breast cancers decreased due to the early detection and advanced treatment recently [2]. However, the complex mechanism of tumorigenesis and development in breast cancer still impede the treatment of this disease. Considering this, more profound mechanism and reliable markers are needed to predict the survival of breast cancer patients.

Dysregulation of posttranscriptional regulation is an important mechanism in the initiation and development of cancer and posttranscriptional mechanism is highjacked to enable swift and robust adjustment of protein expression levels in response to intrinsic and extracellular signals [3, 4]. RNA binding proteins (RBPs) are key players in posttranscriptional events and control the metabolism of RNA targets including transportation, polyadenylation, stability, splicing and degradation by forming different ribonucleoprotein complexes [5–7]. Lots of RBPs have been reported to be dysregulated in cancers and take part in every process of tumor development [8]. RBPs mainly function through their RNA binding domains (RBDs) and are commonly classified based on these RBDs, as the structure and function of these RBDs provide some insights into the binding preferences and RNA targets. Among these RBDs, RNA recognition motif RRM, also known as ribonucleoprotein motif RNP, is the most common and best characterized RBD. The RRM is composed of 80–90 amino acids containing two conserved motif RNP1 and RNP2, which are essential in regulating the metabolism of RNA by binding to AU-rich element (ARE) of mRNA [9]. Accordingly, the RBP proteins, which contain RNA recognition motif RRM domain, are classified into RBM family (RNA-binding motif protein family).

Till now, the role of RBM family in cancer development is less studied. RBM38, a member of RBM family, was found to be dysregulated and acted as tumor suppressor in breast cancer [10]. It inhibited the proliferation of breast cancer by upregulating the expression of PTEN and down regulating the expression of c-Myc [11, 12]. It also increased the stability of ZO-1 (Zonula occludens-1) and inhibited the breast cancer epithelial-mesenchymal transition [10, 13]. RBMS3 was also identified to be a potential treatment target in breast cancer which inhibited the proliferation and invasion of breast cancer [14]. To identify other RBM family members which play a vital role in breast cancer development, TCGA was used to identify these genes and found RBMS2 might be involved in genesis of breast cancer.

RBMS2 is a member of c-Myc single-strand binding proteins (MSSPs) family and encodes RNA binding protein. MSSPs, which contain two conserved RNA binding motifs, RNP1 and RNP2, are absolutely essential for the binding to the c-Myc gene promoter [15] and mRNAs [16] in vitro. In the present study, we examined the hypothesis that RBMS2 is a tumor suppressor in breast cancer. It inhibits the proliferation of breast cancer by regulating the expression of P21 by binding to its 3′-untranslated region (3′-UTR) in a posttranscriptional way.

## Methods

### Cell line and cell culture

The human breast cancer cell lines (MCF-7, ZR-75-1, SK-BR-3, MDA-MB-453 MDA-MB-231 and MCF-10A) were obtained from the American Type Culture Collection (ATCC, USA). The human breast cancer cell line SUM1315 was obtained from Shanghai Institutes of Biological Sciences, Chinese Academy of Science. The cancer cells were cultured in complete high glucose Dulbecco’s modified Eagle medium (DMEM, Wisent, China), supplemented with 10% fetal bovine serum, 100 μg/ml penicillin-streptomycin (Hyclone, USA). The normal breast epithelial cell line MCF-10A was fed with mammary epithelial cell basal medium (MEBM, Lonza, Switzerland) with 100 ng/ml cholera toxin. Cells were cultured in a 5% CO2 cell culture incubator at 37 °C.

### Lentivirus transfection and small interfering RNA

Lentivirus constructed cells were transfected with PGLV3-h1-GFP-puro vector (GenePharma, China) or pGLV5-h1-GFP-puro vector (GenePharma, China) containing either RBMS2 knockdown (shRBMS2) or RBMS2 overexpression (RBMS2), and a scrambled sequence (SCR) or a negative control sequence (NC), respectively, following the manufacturer’s instructions. All plasmids were verified by sequencing (GenePharma, China). Cells were plated in 6 wells dishes at 30% confluence and infected with the retroviruses. Meanwhile, polybrene (5 μg/ml) was added with the retroviruses to enhance the target cells infection efficiency. Stable pooled populations of breast cancer cells were generated by selection using puromycin (2 μg/ml) for 2 weeks.

MCF-7 and SUM-1315 RBMS2 overexpression (RBMS2) and the control (NC) cells were seeded in 6-well plates overnight and then transfected with P21-siRNA (GenePharma, China) and the nonspecific siRNA control, using Lipofectamine® 3000 transfection agent (Invitrogen, USA). The sequences of the siRNAs are as follows:

- sense 5’-CCUCUGGCAUUAGAAUUAUTT-3′ and
- antisense 5’-AUAAUUCUAAUGCCAGAGGTT-3’

### RNA isolation and quantitative RT-PCR analysis

Total RNA was isolated using Trizol reagent (TaKaRa, Japan), and 1000 ng RNA was reverse-transcribed into cDNA using Primescript RT Reagent (TaKaRa, Japan).

The RT–PCR was performed using FastStart Universal SYBR Green Master (Roche, Switzerland) in a real-time PCR instrument (Applied Biosystems, USA), and β-actin was used as endogenous control. The primers listed in Additional file 1: Table S1.

### Western blot analysis

Cells were ruptured with RIPA (Thermo Fisher, USA) buffer containing 1% phosphatase inhibitor, 1% PMSF, and 0.1% protease inhibitor. Cell lysates were resolved by SDS-PAGE and transferred onto PVDF membranes (Millipore, USA). Membranes were blocked for 2 h with 5% skim milk in Tris buffered saline containing 0.1% Tween 20 and incubated overnight at 4 °C with anti-mouse β-actin (Cell Signaling technology, USA), anti-rabbit RBMS2(RayBiotech, USA), anti-rabbit P21(Cell Signaling technology, USA). Membranes were washed 10 min three times with Tris-buffered saline containing 0.1% Tween 20, incubated for 2 h with appropriate secondary antibodies.

### Cell counting kit (CCK-8) assay

Cell proliferation was assessed using CCK-8 kit (Dojindo, Japan) according to the manufacturer’s instructions. 2 × 10^3^ cells suspended in 200 μl medium were seeded in triplicate in a 96-well plate, grown at 37 °C. Medium containing 10% CCK8 replaced the original medium and incubated at 37 °C for 2 h, and the absorbance was finally determined at 450 nm using a micro plate reader.

### Colony formation assay

Cell were cultured into 6-well plates with 500 cells every well and cultured for 15–20 days. When exploring the potential of RBMS2 to function as a tumor suppressor by repressing P21 expression in breast cancer in Fig. 7, cells were cultured for 20 days. The colonies were fixed in 75% absolute ethanol for 60 s, washed twice with PBS and stained with Giemsa (Sigma, USA) for 15 min, then dried at room temperature. The colonies containing 50 or more cells in each well were counted.

### Cell cycle analysis

Cells were collected and fixed in ethanol at − 20 °C overnight. Then cells were washed with PBS, and resuspended in 500 μl of PBS with 0.2% Triton X-100, 10 mM EDTA, 100 μg/ml RNase A, and 50 μg/ml propidium iodide (PI) at room temperature for 30 min. Then cell suspensions were assessed by flow cytometry (Becton Dickinson, San Jose, USA).

### RNA immunoprecipitation (RIP)

RNA immunoprecipitation was carried out as previously described [17]. Cell lysates from SUM-1315 and MCF-7 were prepared with RNA immunoprecipitation lysis buffer (Magna RIP Kit; Millipore, USA) at 4 °C and then incubated with 5 μg of anti-RBMS2 or rabbit IgG overnight at 4 °C. The RNA-protein immunocomplexes were collected by protein A/G magnetic beads, followed by RNA purification. Then PCR and qRT-PCR were used to measure the levels of β-actin and p21 transcripts in the RBMS2 or IgG immunocomplexes.

### RNA stability assays

RBMS2-overexpressing cell lines, and their control cell lines were cultured in 6-well plates. Then actinomycin D (ActD) was added to 5 μg/ml at 0 h, 2 h, 4 h and 8 h before cell scraping collection. Total RNA was isolated and qRT-PCR was performed to quantify the relative levels of P21 as mentioned above.

### Dual-luciferase reporter assay

Dual-luciferase reporter assays were performed using kits (Promega, USA) according to the manufacturer’s instructions. Briefly, 200 ng of a pGL3 reporter containing 3′-UTR region or AREs mutant region of P21 and 5 ng of Renilla luciferase vector (pRL-TK; Promega, USA) as the internal control, were co-transfected into breast cancer cells. The AREs mutant reporter changed the AUUUA motif in P21 3′-UTR region into AGGGA. After 48 h, the cells were harvested to measure the luciferase intensity.

### Breast tissue samples

Eighty breast tissue samples used in immunohistochemical staining (IHC) and analysis were obtained from the First Affiliated Hospital of Nanjing Medical University, China, between 2004 and 2007. The TNM staging was defined according to the American Joint Committee on Cancer (AJCC) (6th version, 2002). Forty-six breast tissue samples used in qRT-PCR were obtained from the First Affiliated Hospital of Nanjing Medical University, China, between 2013 and 2015.

The collection and use of the samples was reviewed and approved by the Institutional Ethics Committee of the First Affiliated Hospital of Nanjing Medical University.

### IHC staining and analysis

IHC staining of the 80 breast tissue samples with RBMS2 (RayBiotech, USA) and P21 (Cell Signaling technology, USA) antibodies was conducted and analyzed. All specimens and implanted tumors were fixed in10% formalin and then embedded in paraffin. After blocking endogenous peroxides and proteins, sections (thickness, 4 μm) were incubated overnight at 4 °C with primary antibodies for specific detection of RBMS2(RayBiotech, USA) or P21 (Cell Signaling technology, USA). After washing with PBS, sections were incubated with HRP-Polymer-conjugated secondary antibody.

at 37 °C for 1 h. Subsequently, sections were stained with 3,3-diaminobenzidine solution for 3 min and the nuclei were counterstained with hematoxylin. The RBMS2 antibody was used at the dilution of 1:100. The P21 antibody was used at the dilution of 1:125. The immuoreactivity scoring system was used to score the staining of breast cancer tissues as described [18]. Overall scores 0–6 were defined as low P21 or RBMS2 expression, and scores > 6 were high expression.

### In vivo tumor xenograft model

All animal experiments were conducted according to the guidelines of Institutional Animal Care and Use Committee of the Nanjing Medical University. Sixteen female BALB/c nude mice (aged 4 weeks, 18–22 g) were randomly allocated to 2 groups. Stable RBMS2-expression SUM-1315 cells or control cells (1 × 10^6^ cells in 0.1 ml PBS) was subcutaneously injected into mammary fat pads of the mice and the growth of tumors was followed up every week. Tumor volume was measured every week using a caliper, calculated as (length × width ×height)/2. After 6 weeks, mice were sacrificed and checked for final tumor weight.

### Cell migration and invasion assay

Cell invasiveness was conducted using 24-well transwell inserts (Millicell Hanging Cell Culture Insert, USA) coated with 50 ml of Matrigel (BD Biosciences, USA). Inserts were seeded with 5 × 10^4^ cells in 200 ml DMEM supplemented with 0.1% FBS, and 500 ml of medium with 10% FBS was added to the lower chamber. Cell migration assay was carried out without the Matrigel. Cells were incubated at 37 °C for 36 h, after which noninvading cells were wiped from the upper side of the membrane. The number of invading or migrating cells was counted under the microscope in five independent fields and the average number of cells per field was represented in the graphs.

### mRNA sequencing

RNA from stable RBMS2-expression SUM-1315 cells or control cells with three duplicates were purified and sent to commercial company to undergo mRNA sequencing (Allwegene, Beijing, China). Differential gene expression was analyzed by standard Illumina sequence analysis pipeline. Foldchange > 2 and FDR (false discovery rate) < 0.05 were used as cut off value to select significant target genes. Gene set enrichment analysis (GSEA) was used to analyze the pathway enrichment in two groups [19, 20]. KEGG pathway was analyzed with David [21] (https://david.ncifcrf.gov/).

### Database analysis

Expression of targeted genes by mRNA Sequencing and clinical data were downloaded from The Cancer Genome Atlas (TCGA) dataset (http://cancergenome.nih.gov/). The expression data between breast cancer tissues and adjacent normal breast tissues were compared by edgeR package in R (version 3.4.1). Foldchange > 2 and FDR < 0.05 were used as cut-off values to select significant target genes. KM-plot website (http://www.kmplot.com/) was used to identify the prognosis of RBMS2 in breast cancer patients in TCGA dataset. The Human Protein Atlas database (https://www.proteinatlas.org/) was used to identify the protein expression of immunohistochemical staining of RBMS2 in breast cancer patients and mRNA expression in breast cell lines [22].

### Statistical analysis

All experiments were repeated in triplicate, unless otherwise specified. The data were analyzed using the SPSS 20.0 software (Chicago, USA). For all the continuous variables, Students t-test was used to analyze the statistical significance of the differences between groups, and *P* < 0.05 was used as a threshold to indicate a statistical significance.

## Results

### RBMS2 was expressed lower in human breast cancer cells and tissues

**Fig. 1:**
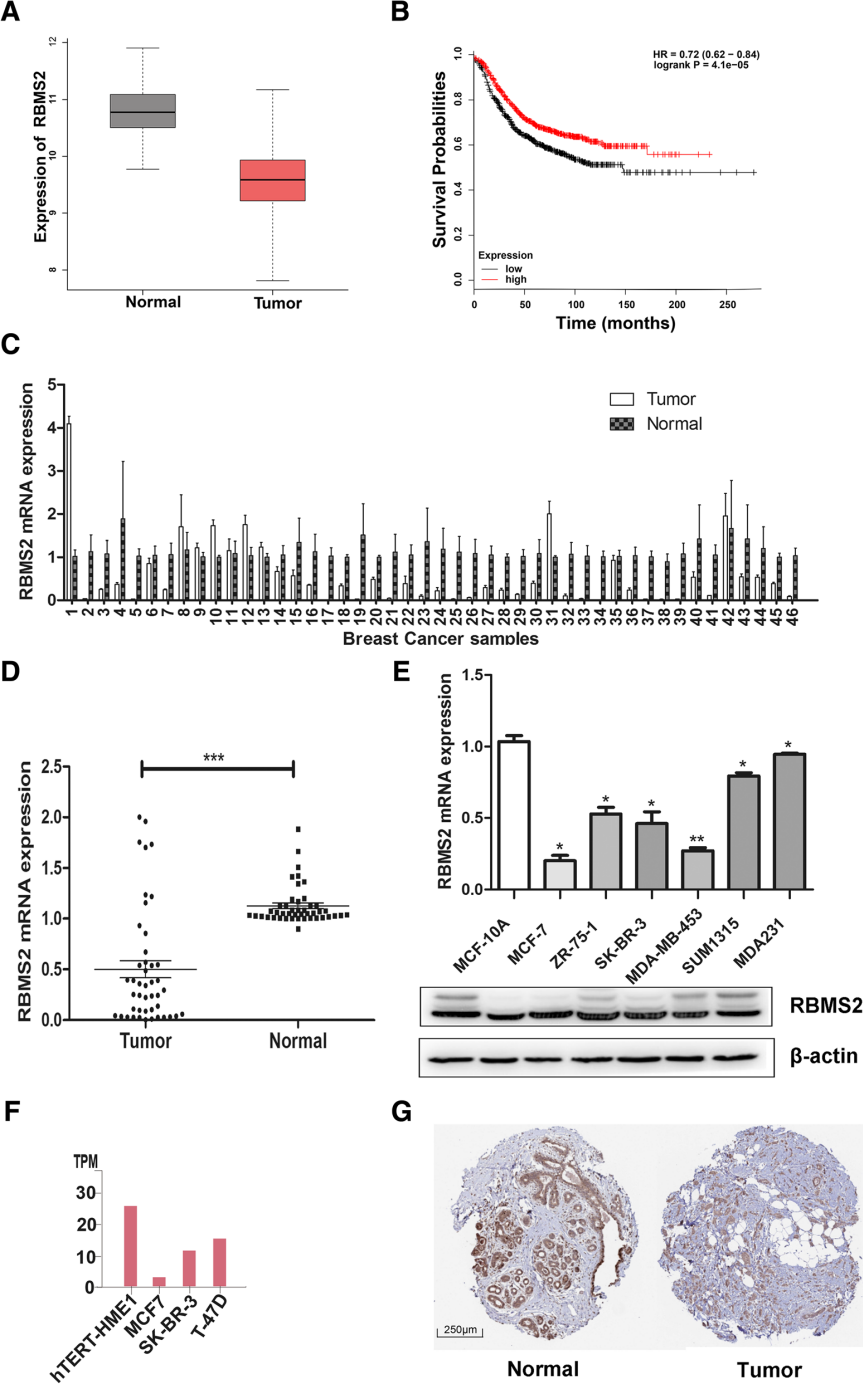
RBMS2 expression in breast cancer tissues. **a** Comparison of RBMS2 transcript expression in tumor and normal tissues of breast cancer in the Cancer Genome Atlas (TCGA) database (*p* < 0.01). The expression values were log2-transformed. **b** Kaplan-Meier survival curves of RFS based on RBMS2 expression in breast cancer using Kaplan-Meier plotter. The cut-off value was the median expression of RBMS2. **c** RBMS2 mRNA expression in 46 pairs of breast cancer and adjacent tissues. **d** Average expression level of RBMS2 mRNA in 46 pairs of human breast cancer tissues and adjacent normal breast tissues. Adjacent breast tissues had higher expression of RBMS2 than breast cancer tissues (***p < 0.001). **e** qRT-PCR (upper) and western blot (lower) of RBMS2 in breast cancer cell lines and in normal breast cell lines MCF-10A. The average level of mRNA in MCF-10A was set as 1. **f** RBMS2 mRNA in breast cancer cell lines and in normal breast cell lines hTERT-HME1 in the Human Protein Atlas database. Expression quantification was shown as TPM (Transcripts Per Million). **h** Representative IHC stains of RBMS2 in normal and tumor breast tissues in the Human Protein Atlas database. Scale bars indicated 250 μm

To identify RBM family members which play important roles in breast cancer, TCGA database was used to discover the differently expressed genes in breast cancer tissues and normal tissues. All the differently expressed genes in breast cancers were listed in Additional file 2: Table S2. We found RBMS2, RBMS3, RBMXL2 were the significantly differently expressed RBMs in breast cancer. RBMXL2 had a very low expression in both breast tissue and cancer in TCGA database (Additional file 3: Figure S1) and RBMS3 was reported to be a tumor suppressor in breast cancer [14]. RBMS2 was significantly down expressed in breast cancer with log (foldchange) -1.21 and FDR < 0.05(Fig. 1a). KM plot was further used to explore the relationship of RBMS2 expression with survival of breast cancer patients. In RFS, RBMS2 was a favorable factor for breast cancer patients with HR (0.72 (0.62–0.84), *p* < 0.05, Fig. 1b) t. We further confirmed that RBMS2 was expressed lower in breast cancer tissues than adjacent normal tissues of patients in our hospital. The expression of RBMS2 was also lower in breast cancer cells compared to normal breast cell line (MCF-10A, Fig. 1e) both in mRNA and in protein. It accords with mRNA sequencing data of cell lines from the Human Protein Atlas database (Fig. 1f). IHC (Fig. 1g) from the Human Protein Atlas database confirmed the lower expression of RBMS2 in breast cancer in protein level. Moreover, we analyzed the correlation between the expression of RBMS2 and clinicopathological characteristics of breast cancers in 80 patients. As shown in Table 1, the tumor sizes of breast cancers that have high expression of RBMS2 are smaller than that of those who have low expression.

**Table 1:** Expression of RBMS2 expression in human breast cancer according to patients’ clinicopathological characteristics

Characteristics	RBMS2		*p*-value
	High group	Low group	
Tumor Size			
< 2	21	11	0.01543
≥ 2	17	31	
Stage			
I/II	34	36	0.6115
III/IV	4	6	
Lymph node metastasis			
Present	36	33	0.07646
Absent	2	9	
Age			
< 50	15	20	0.6116
≥ 50	23	22	
P21			
Low	10	24	0.0105
High	28	18	

### RBMS2 inhibited tumor proliferation and induced cell cycle arrest

**Fig. 2:**
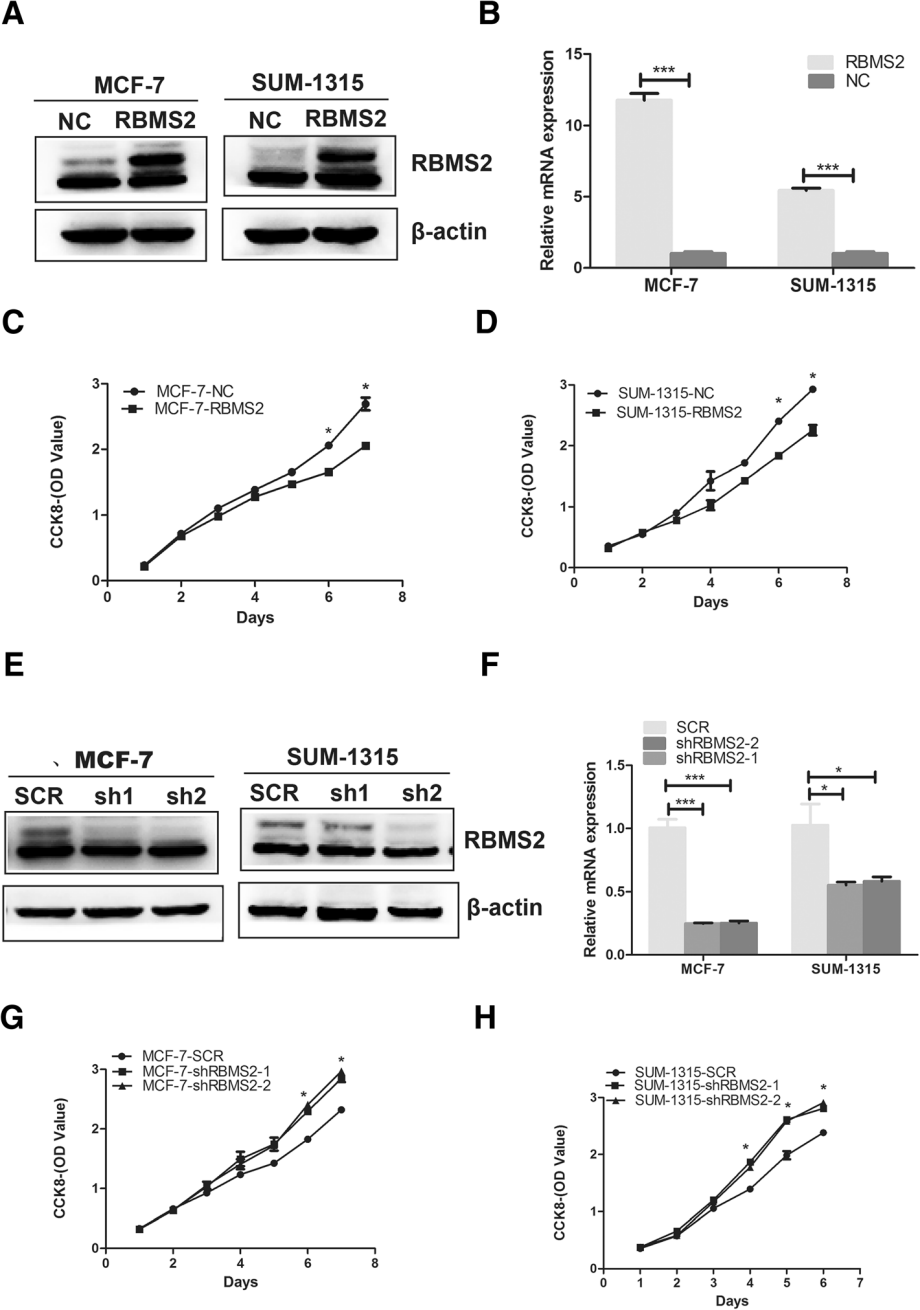
Construction of RBMS2 overexpression and knockdown cell lines. Western blot (**a**) and qRT-PCR (**b**) were used to confirm RBMS2 overexpression after tansfection with lentivirus in MCF-7 and SUM-1315 cell lines. The upper band of RBMS2 is the specific band in western blot. NC, control; RBMS2, lentivirus overexpressing RBMS2; The growth of cells was measured using CCK8 assays in RBMS2 overexpressing MCF-7 (**c**) and SUM-1315 (**d**) cell lines. Western blot (**e**) and qRT-PCR (**f**) were used to confirm RBMS2 knockdown after transfection with lentivirus in MCF-7 and SUM-1315 cell lines. SCR, control; shRBMS2, lentivirus knocking down RBMS2. The growth of cells was measured using CCK8 assays in RBMS2 knockdown MCF-7 (**g**) and SUM-1315 (**h**) cell lines

**Fig. 3:**
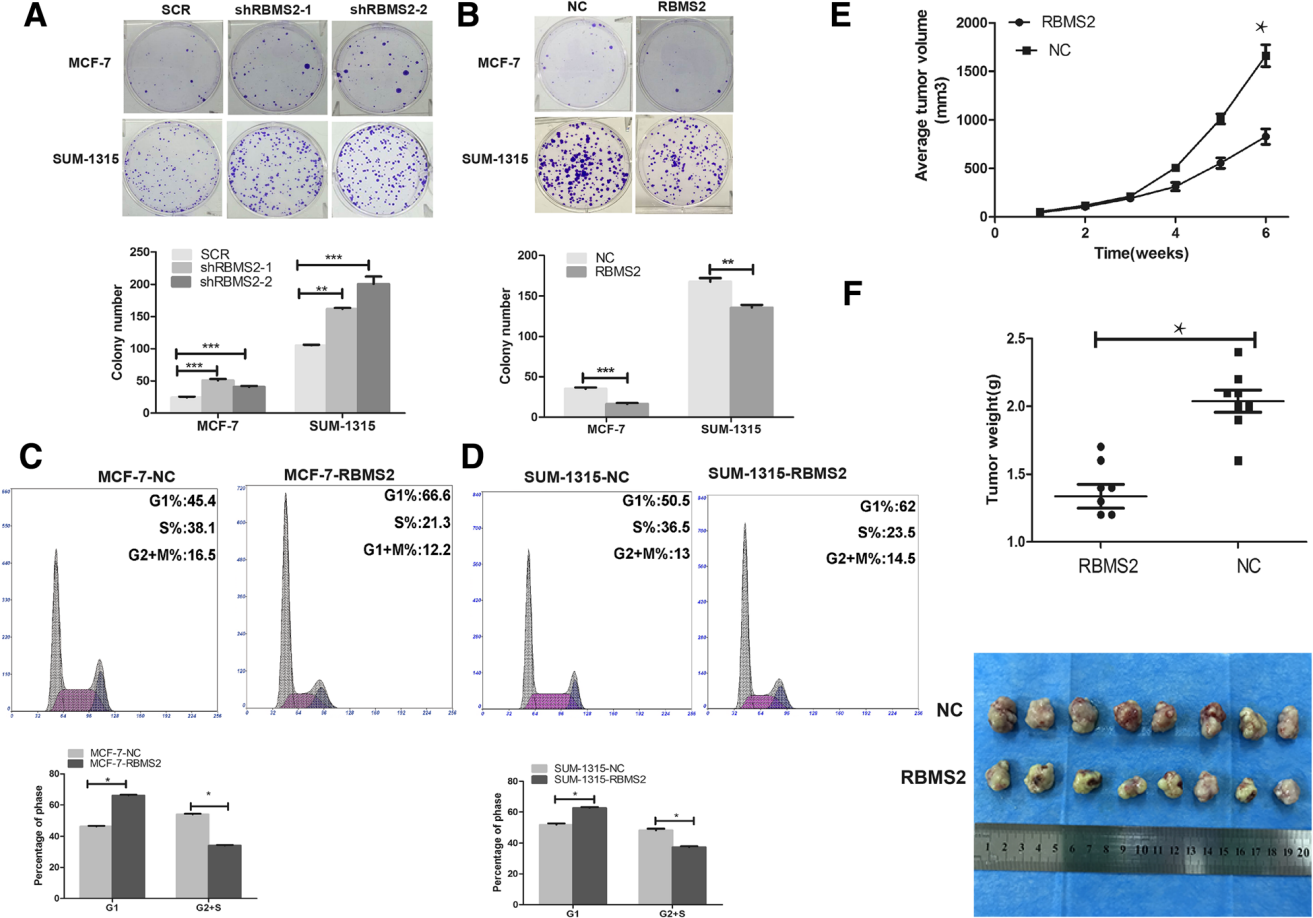
Effect of RBMS2 on proliferation and growth of breast cancer cell lines MCF-7 and SUM-1315 in vitro and*in vivo*. The growth of cells was measured using colony formation assays in RBMS2-overexpressing MCF-7 and SUM-1315 cell lines. Representative photographs (upper) and quantification (lower) were shown.(**p < 0.01,***p < 0.001). The growth of cells was measured using colony formation assays in RBMS2-knockdown MCF-7 and SUM-1315 cell lines. Representative photographs (upper) and quantification (lower) were shown (**p < 0.01,***p < 0.001). Cell cycle progression was measured using flow cytometry in RBMS2-overexpression MCF-7 (**c**) and SUM-1315 (**d**) cell lines. Representative photographs (upper) and quantification (lower) were shown. (*p < 0.05) (**e**) Tumor volume in RBMS2-overexpressing SUM-1315 compared with control in nude mice in different time points (**f**) Tumor weight in RBMS2-overexpressing SUM-1315 compared with control in nude mice at the end point. Quantifiaction (upper) and representative photographs (lower) were shown

To explore the role of RBMS2 in proliferation, MCF-7 and SUM-1315 were chosen to transfect with RBMS2 overexpression or knockdown Lentivirus based on the expression of RBMS2 in different cell lines. Western blot (Fig. 2a and e) and qRT-PCR (Fig. 2b and f) were used to confirm the transfection efficiency. Different siRNAs may act differently in different cell lines. Actually, sh2 acted better than sh1 in our study. But sh1 still knocked down most of the expression of RBMS2. The effect of RBMS2 on proliferation was then evaluated with CCK-8 assay. As shown in Fig. 2c-d, RBMS2 overexpression inhibited the proliferation of MCF-7 and SUM-1315 cell lines, while knockdown of RBMS2 promoted the proliferation of these two cell lines (Fig. 2f-g). In addition, colony formation assay was used to evaluate the effect of RBMS2 on anchorage-independent growth. The results revealed that the capability of RBMS2 overexpression cells to form colonies was much poorer than that of control cells (Fig. 3a). Also, knockdown of RBMS2 increased the ability to form colonies (Fig. 3b). The impact of cell cycle arrest induced by RBMS2 was evaluated by flow cytometry. As shown in Fig. 3c-d, RBMS2 overexpression cells induced cells to arrest in G1 phase both in MCF-7 and SUM-1315 cell lines. Migration and invasion assays didn’t show RBMS2 affect migration and invasion of breast cancer (data not shown).

### RBMS2 inhibited tumorigenesis in nude mice

To evaluate the effects of RBMS2 in tumor suppression in vivo, SUM-1315 cells which were transfected with RBMS2 overexpression and control lentivirus were injected into mammary fat pads of the mice. As shown in Fig. 3e-f, tumor volume of RBMS2 overexpression group increased slower than that of control group. Six weeks later, tumor weights in RBMS2 overexpression group were significantly lower than that of control group, which indicates RBMS2 inhibited tumor proliferation in vivo.

### RBMS2 regulated the expression of P21 in breast cancer cells

**Fig. 4:**
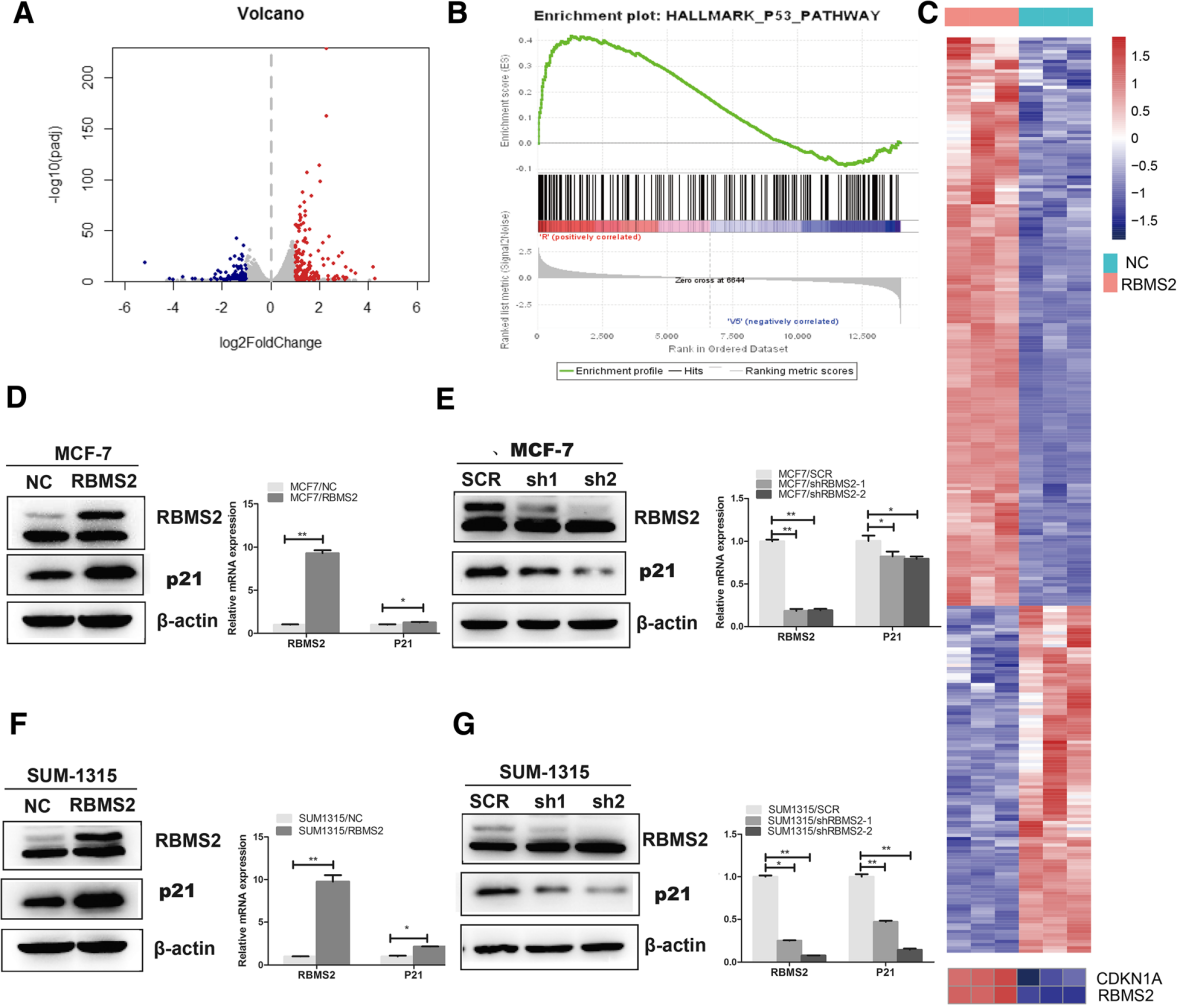
Differential expression genes and pathways regulated by RBMS2. **a** Total 285 genes were differentially identified from mRNA sequencing. Among them, 177 genes (red) were up regulated and 108 genes (blue) were down regulated. **b** Gene Set Enrichment Analysis (GSEA) of differentially expressed genes. **c** Heatmap of differentially expressed genes from mRNA sequencing (upper). Heatmap of expression of CDKN1A and RBMS2 from mRNA sequencing (down).Sky blue indicated RBMS2 overexpression cell lines and red indicates control cell lines. The MCF7 cells were transfected with lentivirus to overexpress RBMS2 (**d**) or knock down RBMS2 (**e**). The P21 expression was obviously increased after upregulating RBMS2 at both protein and mRNA levels (**d**). The P21 expression was obviously decreased after knocking down RBMS2 at both protein and mRNA levels (**e**). Western blot (left) and mRNA (right) photographs were shown. The SUM-1315 cells were transfected with lentivirus to overexpress RBMS2 (**f**) or knock down RBMS2 (**g**). The P21 expression was obviously increased after upregulating RBMS2 at both protein and mRNA levels (**f**). The P21 expression was obviously decreased after knocking down RBMS2 at both protein and mRNA levels (**g**). Western blot (left) and mRNA (right) photographs were shown

To identify potential mRNA targets regulated by RBMS2, RNA sequencing was performed in SUM-1315 cell. Three overexpressing and three control stable cell lines were selected for RNA sequence. Total 285 genes were differentially identified from mRNA sequencing (Fig. 4a). Among them, 177 genes were upregulated and 108 genes were downregulated. All genes are listed in Additional file 4: Table S3. Two pathways were identified significantly (Table 2) and the P53 pathway was highly related to cancer development, which was also evaluated by GSEA in Fig. 4b. In these genes of P53 pathway, P21 (CDKN1A) was significantly upregulated after overexpression of RBMS2 with log2 (foldchange) 1.3 and FDR < 0.05(Fig. 4c).

**Table 2:** KEGG pathway analysis

Pathway	Gene number	*p*-value
Pathways in cancer	18	0.035
Cytokine-cytokine receptor interaction 13	13	0.032

Moreover, the expression of P21 was significantly upregulated upon the overexpression of RBMS2 in MCF-7 (Fig. 4d) and SUM-1315 (Fig. 4f) cell lines in both qRT-PCR and western blot. Accordingly, there was a significant decrease of P21 in protein and mRNA level upon knockdown of RBMS2 (Fig. 4e, g).

**Fig. 5:**
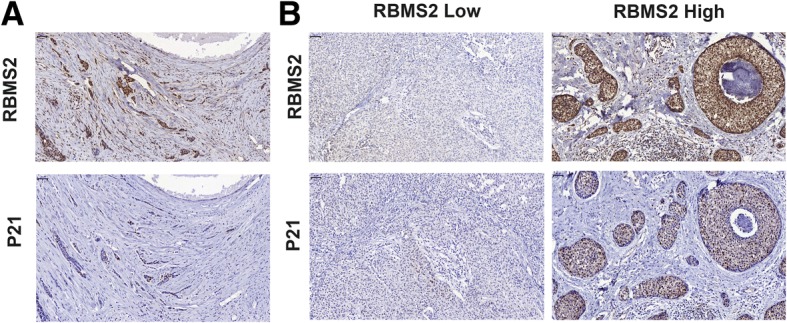
RBMS2 expression correlated positively with P21 in human breast cancer tissues. **a** IHC analysis of RBMS2 and P21 expression in breast cancer at 400× magnification. RBM38 and P21 were expressed in the cytoplasm and nucleus. Scale bars indicated 50 μm. **b** IHC analysis of RBMS2 and P21 expression in breast cancer at 400× magnification. The breast cancer with the high staining of P21 expressed high level of RBMS2; the breast cancer with the low staining of P21 expressed low level of RBMS2. Scale bars indicated 50 μm

IHC staining was also confirmed that the expression of P21 was positively correlated with RBMS2 (Fig. 5a-b, Table 1). In TCGA database, P21 and RBMS2 mRNA was also positively correlated in breast cancer petients (Additional file 5: Figure S2).

### RBMS2 enhanced P21 stability by directly binding to the AREs in the 3′-UTR of P21 mRNA

**Fig. 6:**
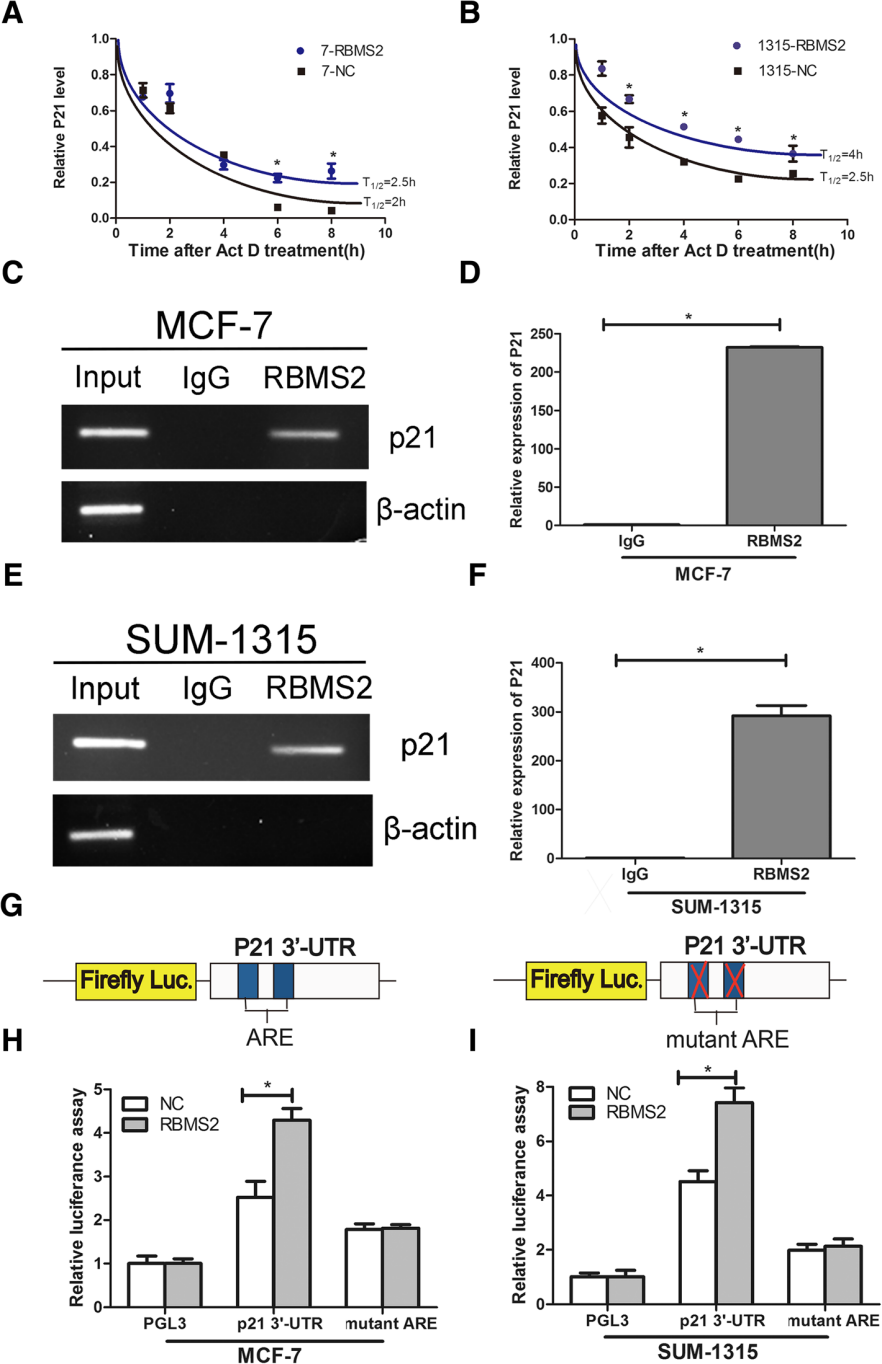
RBMS2 promoted mRNA stability by directly binding to P21 mRNA 3′-UTR. The relative expression of P21 in RBMS2-overexpressing MCF-7 (**a**) and SUM-1315 (**b**) cell lines after treated with 5 μg/ml ActD for 0, 2, 4, 6, and 8 h. MCF-7 (**c** and **d**) and SUM-1315 (**e** and **f**) cell lysates were collected and immunoprecipitated with RBMS2 antibody or control IgG followed by PCR (**c** and **e**) and qRT–PCR (**e** and **f**) measuring transcript levels of P21 in the control or RBMS2-mRNA immunocomplexes. **g** Dual-luciferase reporter containing P21 3′-UTR region (left) and AREs mutant region were constructed (right). Relative luciferase activity of P21–3′- UTR or mutant ARE after co-transfection with RBMS2-overexpressing (RBMS2) or control vector (Ctrl) into MCF-7 (**h**) and SUM-1315 (**i**) cell lines. Firefly luciferase activity was measured and normalized to Renilla luciferase activity. ARE, AU-rich element

RNA binding proteins often posttranscriptionally regulate mRNA stability. To investigate whether RBMS2 regulate the expression of P21 in a posttranscriptional way, RBMS2 overexpression and control cells were treated with 5 mg/ml ActD at different time points in both MCF-7 and SUM-1315 cells lines. In MCF-7 cell, RBMS2 overexpression cells showed a longer half-life of 2.5 h, compared with 2 h in control cells (Fig. 6a). Similar results were found in SUM-1315 cell line (Fig. 6b), in which RBMS2 overexpression cells processed a longer half-life of 4 h. This indicated that RBMS2 enhanced the stability of P21 mRNA in a posttranscriptional manner. To further investigate whether RBMS2 directly binds to the P21 mRNA, RIP assay was performed in MCF-7(Fig. 6c-d) and SUM-1315(Fig. 6e-f) cell lines. The P21 mRNA was detected in RBMS2 antibody elution rather than IgG elution in both PCR and qRT-PCR, which indicated the direct binding of RBMS2 to P21 mRNA. β-actin which served as a negative control was not bound by RBMS2. In Fig. 6d-f, mRNA in RBMS2 antibody elution is much more than that in IgG elution using RT-PCR. We also performed a dual-luciferase assay using reporters that carried the entire region of P21 3′-UTR or AREs mutant P21 3′-UTR to confirm that RBMS2 regulated P21 through binding to the AREs in 3’-UTR of P21 mRNA (Fig. 6g). RBMS2 increased the luciferase activity of a reporter carrying the 3′ -UTR of the p21 mRNA compared with control in MCF-7 and SUM-1315 cell lines. The luciferase activity of a reporter carrying ARE sites mutant P21 3′-UTR showed no difference between RBMS2 overexpression and control group, which proved that RBMS2 increased the stability of P21 dependent of ARE sites of P21 3′-UTR (Fig. 6h-i).

### Specific inhibition of P21 reversed the suppression of proliferation induced by RBMS2 overexpression

**Fig. 7:**
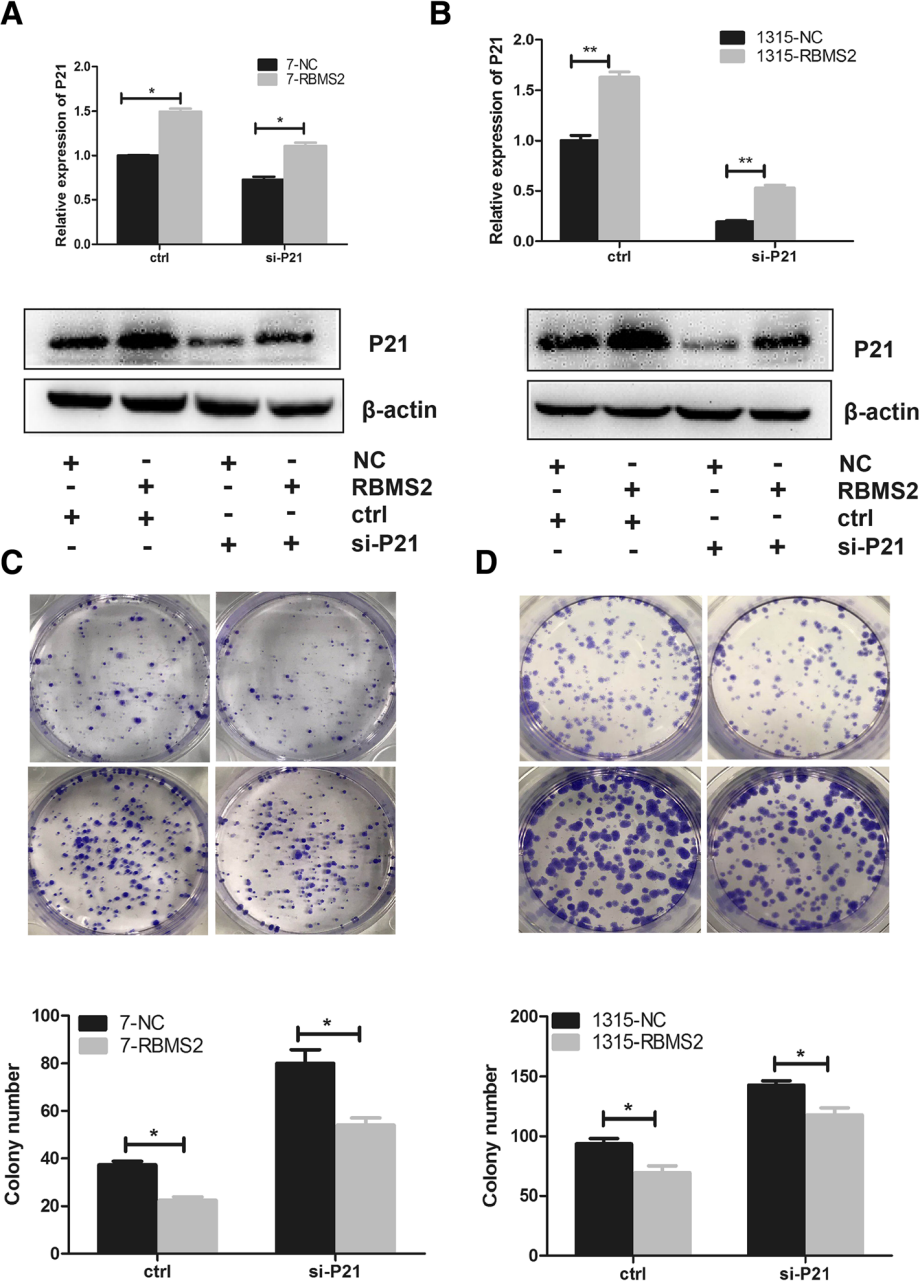
Anti-proliferation activity induced by RBMS2 overexpression was rescued by small interfering P21. RBMS2-overexpressing and control MCF-7 (**a**) and SUM-1315 (**b**) cell lines were transfected with small interfering P21. Western blot and RT-PCR were used to confirm the efficiency. RT-PCR (upper) and western blot (down) photographs are shown. The growth of MCF-7 (**c**) and SUM-1315 (**d**) cells mentioned above was measured using colony formation assays. Cells cultured for 20 days. Representative photographs (upper) and quantification (lower) were shown

To explore the potential of RBMS2 to function as a tumor suppressor by repressing P21 expression in breast cancer, small interfering RNA or control was transfected into RBMS2 overexpression SUM-1315 and MCF-7 cells. The transfection efficiency was confirmed by qRT-PCR and western blot (Fig. 7a-b). In colony formation assay, the average colony numbers decreased after overexpression of RBMS2. The colony numbers were rescued after we interfered the expression of P21 (Fig. 7c-d).

## Discussion

Aberrant expression of RBPs plays a vital role in the initiation and development of cancer [7]. In TCGA database, RBMS2 and RBMS3 were both down regulated, while RBMXL2 was upregulated. The opposing results among the RBM family members may be due to the deviations caused by the difference in their sequences, domains and/or cellular functions. RBMS3 was reported to have a low expression in breast cancer tissues and cell lines and inhibit the proliferation and metastasis of breast cancer [14] and RBMXL2 was mainly expressed in testis tissue while it had a very low expression in both breast tissue and cancer in Human Protein Atlas database. In the present study, the role of RBMS2 was studied in breast cancer and we found that it acted as a novel tumor suppressor by stabilizing P21 mRNA in breast cancer. RBMS2 was found to be downregulated in breast cancer both from TCGA databases analysis and the patients’ samples from our hospital. Moreover, RBMS2 was a favorable factor for breast cancer patients. RBMS2 was proved to inhibit the proliferation of breast cancer. Overexpression of RBMS2 inhibited the proliferation breast cancer while knockdown of RBMS2 promoted it both in vivo and in vitro. To clarify the mechanisms underlying the effects of RBMS2 in breast cancer proliferation, mRNA sequencing using overexpression RBMS2 and control SUM-1315 cell lines was performed. RNA sequencing and the following pathway analysis were used to identify the main targets regulated by RBMS2. P53 pathway was reported as an important pathway in different cancers and genes in this pathway can regulate the proliferation of cancer. In our study, P53 pathway was found as the main pathway regulated by RBMS2 and P21 as an important part in P53 pathway was significantly upregulated after overexpressing RBMS2. P21, also known as cyclin-dependent kinase inhibitor 1 (CDKN1A), is a direct target of P53 and mediates G1 growth arrest in response to various stimuli [23, 24]. P21 promotes anti-proliferative activities mainly by binding to and inhibiting various cell cycle proteins, such as CDK2 which leads to growth arrest at specific stages in the cell cycle. P21 was reported down regulated in many cancers [25–27] and the low expression of P21 was associated with large tumor size. High histological grade of P21 expression was related to short disease-free survival (DFS) in breast cancer patients. In our study, RBMS2 positively regulated the expression of P21 and anti-proliferation activity induced by overexpression of RBMS2 was rescued by interfering the expression of P21, which indicates P21 was a main target of RBMS2 in its promoting its anti-proliferation activity in breast cancer. MSSPs family member RBMS1 was reported to regulate c-Myc but no such function has been described for RBMS3 and RBMS2. In our RNA-sequence results, RBMS2 do not regulate the expression of c-MYC. So we can’t prove that RBMS2 functions through c-MYC in breast cancer and more experiments are needed to prove it.

Posttranscriptional regulation is a common way in regulating the expression of P21 in cancer development. Many RBPs have been reported to regulate P21 mRNA stability [28–30]. ELAVL1(ELAV Like RNA Binding Protein 1) enhances the stability of P21 mRNA through binding to 3′-UTR of its transcript upon exposure to UVC [31, 32]. RBM38(RNA Binding Motif Protein 38) also stabilizes P21 transcript by binding to the AU-rich elements in the 3′-UTR [33]. The conserved RNA binding motifs of RBMS2, RNP1 and RNP2, are considered to interact with mRNA. It was reported that RBMS1 [34] and RBMS3 [15] interact with AU-rich element in 3’UTR. RBMS3 regulates the stability of SMAD2(SMAD Family Member 2) mRNA by binding to its 3′-UTR [35]. RBMS3 also mediates the expression of PTF1A (Pancreas Specific Transcription Factor, 1a) by binding to its mRNA 3′-UTR during mouse pancreas development [36]. In the present study, we found RBMS2 increased the mRNA stability of P21 in a transcriptional way by binding to the AU-rich element of 3′-UTR region. It indicates RBMS2 regulated cancer related genes in a posttranscriptional way.

## Conclusions

In summary, our study identified those RBM family members which play important roles in breast cancer development and cancergenesis. RBMS2 as a member of RBM family acts as a tumor suppressor in breast cancer and our study highlighted the role of inhibiting tumor proliferation. P21 as a direct role of RBMS2 indicated the way in which RBMS2 regulated targeted genes and the main pathways. This understanding of RBMS2 in breast cancer proliferation will help in the development of new breast cancer therapeutic approaches.

## Additional files

Additional file 1: **Table S1.** The primers used in quantitative RT-PCR. (DOC 27 kb)

Additional file 2: **Table S2.** Differential expressed genes in breast cancer from TCGA database. (XLS 612 kb)

Additional file 3: **Figure S1.** Expression of RBMXL2 in different cancers in TCGA from the Human Protein Atlas database. ACC: Adrenocortical carcinoma; BLCA: Bladder Urothelial Carcinoma; BRCA: Breast invasive carcinoma; CESC: Cervical squamous cell carcinoma and endocervical adenocarcinoma; CHOL: Cholangiocarcinoma;COAD: Colon adenocarcinoma; DLBC: Lymphoid Neoplasm Diffuse Large B-cell Lymphoma; ESCA: Esophageal carcinoma; GBM: Glioblastoma multiforme; HNSC: Head and Neck squamous cell carcinoma; KICH: Kidney Chromophobe; KIRC: Kidney renal clear cell carcinoma; KIRP: Kidney renal papillary cell carcinoma; LAML: Acute Myeloid Leukemia; LGG: Brain Lower Grade Glioma; LIHC: Liver hepatocellular carcinoma; LUAD: Lung adenocarcinoma; LUSC: Lung squamous cell carcinoma; MESO: Mesothelioma; OV: Ovarian serous cystadenocarcinoma; PAAD: Pancreatic adenocarcinoma; PCPG: Pheochromocytoma and Paraganglioma; PRAD: Prostate adenocarcinoma; READ: Rectum adenocarcinoma; SARC: Sarcoma; SKCM: Skin Cutaneous Melanoma; STAD: Stomach adenocarcinoma; TGCT: Testicular Germ Cell Tumors; THCA: Thyroid carcinoma; THYM: Thymoma; UCEC: Uterine Corpus Endometrial Carcinoma; UCS: Uterine Carcinosarcoma; UVM: Uveal Melanoma; T: Tumor; N: Normal (TIF 992 kb)

Additional file 4: **Table S3.** Differential genes identified from mRNA sequencing. (XLS 24 kb)

Additional file 5: **Figure S2.** Correlation of RBMS2 and P21 mRNA in breast cancer in TCGA database. (TIF 618 kb)

## Abbreviations

3′-UTR: 3′-untranslated region
ActD: Actinomycin D
FDR: False discovery rate
IHC: Immunohistochemical staining
MSSPs: c-Myc single-strand binding proteins
RBDs: RNA binding domains
RBM: RNA-binding motif protein family
RBPs: RNA binding proteins
RIP: RNA immunoprecipitation
ZO-1: Zonula occludens-1
