# Supplementary material for: RBMS2 inhibits the proliferation by stabilizing P21 mRNA in breast cancer
Source: J Exp Clin Cancer Res. 2018 Dec 4;37:298. doi: 10.1186/s13046-018-0968-z (PMC6278172; doi:10.1186/s13046-018-0968-z)
Supplement: Supplementary file 1 — Table S1. The primers used in quantitative RT-PCR. (DOC 27 kb) [file 13046_2018_968_MOESM1_ESM.doc]

**Supplementary Table 1: The primers used in quantitative RT-PCR**

| β-actin | Forward | 5′-GCTGTGCTATCCCTGTACGC-3′ |
| --- | --- | --- |
| β-actin | Reverse | 5′-TGCCTCAGGGCAGCGGAACC-3′ |
| RBMS2 | Forward | 5′-AGTTCTGACACCAGGGATGG-3′ |
| RBMS2 | Reverse | 5′-TGCTCCTCGACTGAAACA-3′ |
| p21 | Forward | 5′-TGTCCGTCAGAACCCATGC-3′ |
| p21 | Reverse | 5′-AAAGTCGAAGTTCCATCGCTC- 3′ |
