# Supplementary figures and images for: RBMS2 inhibits the proliferation by stabilizing P21 mRNA in breast cancer
Source: J Exp Clin Cancer Res. 2018 Dec 4;37:298. doi: 10.1186/s13046-018-0968-z (PMC6278172; doi:10.1186/s13046-018-0968-z)

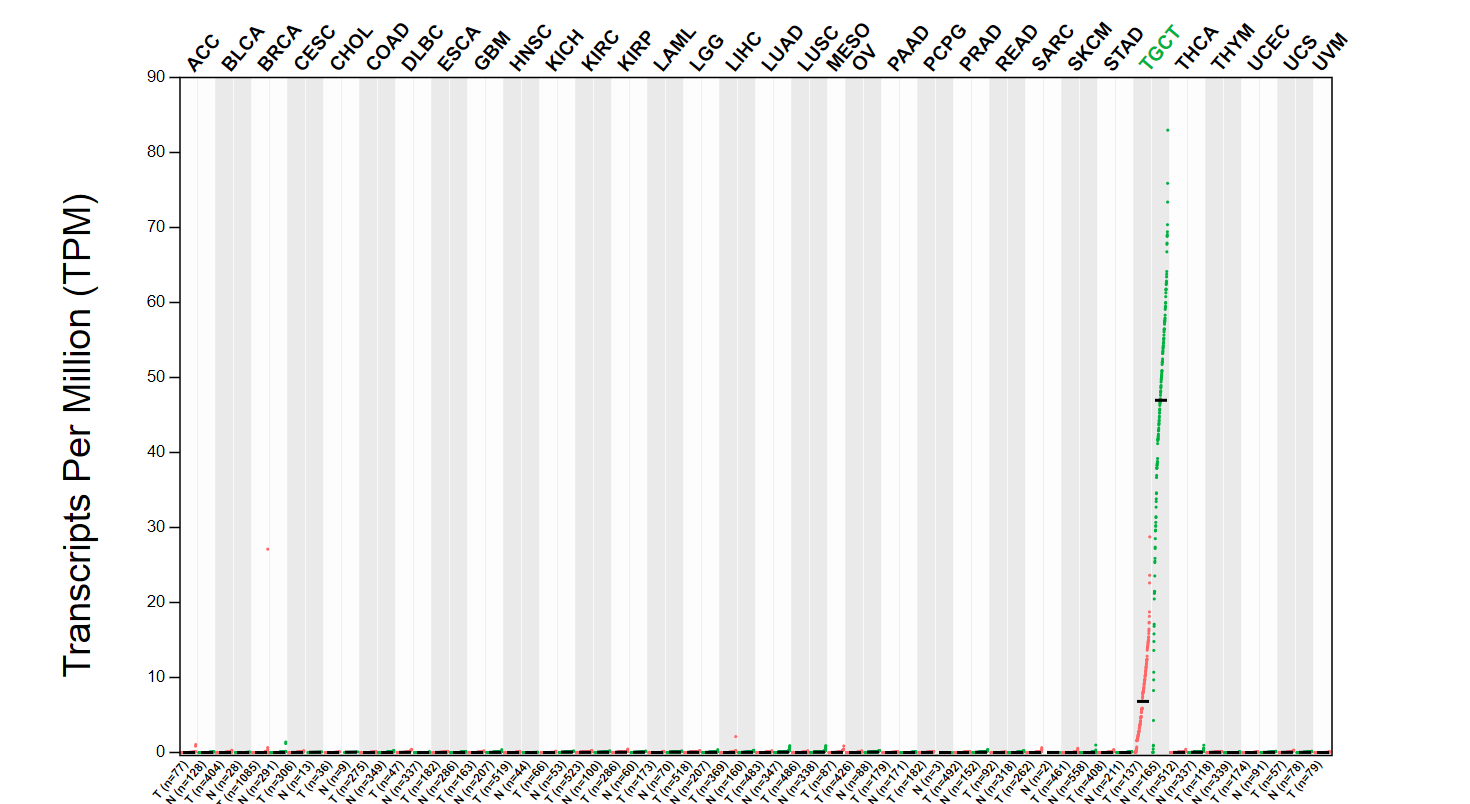

Supplement: Supplementary file 3 — Figure S1. Expression of RBMXL2 in different cancers in TCGA from the Human Protein Atlas database. ACC: Adrenocortical carcinoma; BLCA: Bladder Urothelial Carcinoma; BRCA: Breast invasive carcinoma; CESC: Cervical squamous cell carcinoma and endocervical adenocarcinoma; CHOL: Cholangiocarcinoma;COAD: Colon adenocarcinoma; DLBC: Lymphoid Neoplasm Diffuse Large B-cell Lymphoma; ESCA: Esophageal carcinoma; GBM: Glioblastoma multiforme; HNSC: Head and Neck squamous cell carcinoma; KICH: Kidney Chromophobe; KIRC: Kidney renal clear cell carcinoma; KIRP: Kidney renal papillary cell carcinoma; LAML: Acute Myeloid Leukemia; LGG: Brain Lower Grade Glioma; LIHC: Liver hepatocellular carcinoma; LUAD: Lung adenocarcinoma; LUSC: Lung squamous cell carcinoma; MESO: Mesothelioma; OV: Ovarian serous cystadenocarcinoma; PAAD: Pancreatic adenocarcinoma; PCPG: Pheochromocytoma and Paraganglioma; PRAD: Prostate adenocarcinoma; READ: Rectum adenocarcinoma; SARC: Sarcoma; SKCM: Skin Cutaneous Melanoma; STAD: Stomach adenocarcinoma; TGCT: Testicular Germ Cell Tumors; THCA: Thyroid carcinoma; THYM: Thymoma; UCEC: Uterine Corpus Endometrial Carcinoma; UCS: Uterine Carcinosarcoma; UVM: Uveal Melanoma; T: Tumor; N: Normal (TIF 992 kb) [file 13046_2018_968_MOESM3_ESM.tif]

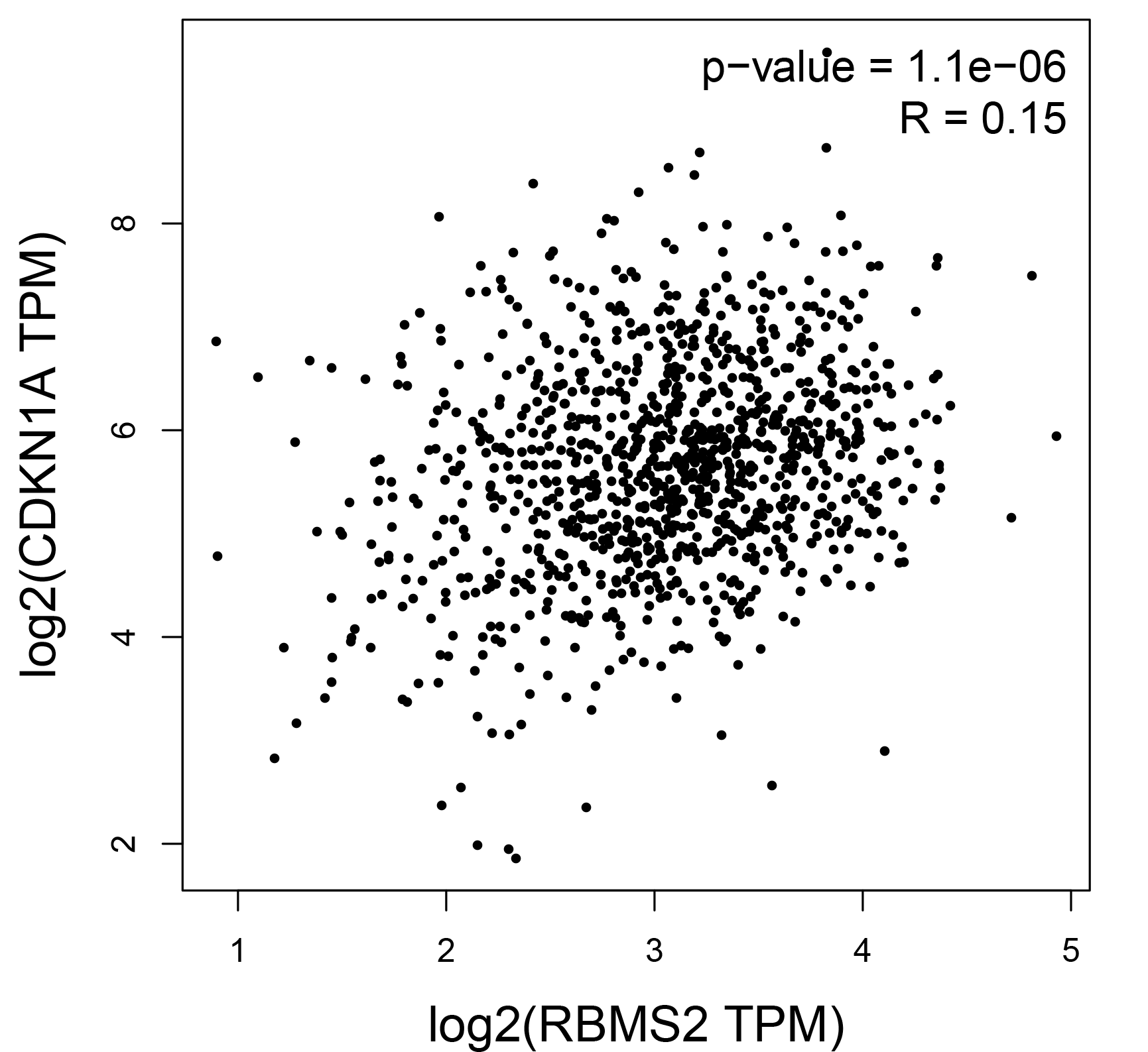

Supplement: Supplementary file 5 — Figure S2. Correlation of RBMS2 and P21 mRNA in breast cancer in TCGA database. (TIF 618 kb) [file 13046_2018_968_MOESM5_ESM.tif]
